# Watt and William’s Base

**Published:** 1870-10

**Authors:** 


### WATT AND WILLIAMS' BASE.

Having had considerable experience in working this recently discovered valuable metal as a base for artificial teeth, and not seeing any mention of the manner of working it in any of the Dental journals, I herewith venture a short history of my experience.

I take two impressions; one of which I fill with plaster, for getting up the trial plate, and as part of the articulator for grinding the teeth; the second I fill with equal parts of plaster and pulverized soapstone, to prevent cracking in drying out. If a chamber is required, it should be cut in this impression.

Trial plates.—If gutta percha is used, heat only to soften. Too much heat makes it sticky, and the borders of the plate are more liable to turn up and leave the cast. Tin or lead, a little thicker than the required plate, is better; either, with care, may be burnished to fit the plaster cast, and in my opinion no time is lost in getting up a zinc die from the plaster model, after the teeth have been ground in, for the plate can be burnished and waxed up, so as to require no filing or scraping. These plates need only be large enough to extend under the teeth far enough for attachment of the teeth.

In waxing up the job, use only enough to make the plate as large and full as it is desired when in the mouth.

The work being now ready for investing, using, of course, Geo. Watt & Co.'s new flask, we take the part having the guides on, placing it on a level surface, having previously prepared the plaster and soapstone (equal parts), and fill in a little above the open spaces of the flask, and place in the cast and fill up the flask; having the wax rim of the plate a little above the edges of the flask, which we cover with the soft plaster. When hard enough, trim so the flasks fit together, that the two parts will separate easily, form the gutter for pouring in the metal from the heel of the plate to one of the openings in the flask. The piece is now varnished and oiled, the two parts of the flask placed together and filled with the same material as the other part. On separating the cast the teeth will be in the upper part of the flask and the cast in the lower.

The trial plate and wax are now removed. Before separating the flask immerse it in a vessel of water, and heat up until the hand can scarcely be borne in it. The wax will then be easily removed.

In the upper part of the flask will be found a surplus of plaster corresponding with the groove cut for pouring the metal. This must be trimmed out to correspond with the lower, and a vent from the opposite heel of the plate. I also cut a large hole in the plaster back of the plate (in upper sets), as a reservoir for the air, from which I make several slight grooves from the edges of the plate, with one to the edge of the flask, that the air may not be confined and prevent the plate from becoming perfect. I also use the same precaution at the heels of the plate, both inside and out (from every place where the air can not escape from the regular vent). If the wax is not thoroughly removed, place in boiling water a few minutes, washing the bits of plaster from under the teeth with warm water. The flask is then confined together and dried out perfectly, which can be done in from one hour to an hour and a half. And in all cases have the metal ready for pouring, when you take the flask from the fire, as it pours much sharper; and in pouring fill the neck of the flask partly full to force the air from the cast into the reservoir. After taking the plate from the flask, if there is any deficiency or holes in it from little particles of plaster being broken from the investment, they can be remedied by soldering with the same material, using as a flux a solution of the chloride of zinc, with a common blow pipe, taking the precaution to cover the opposite side with plaster and solder while green. After filing and scraping to the desired shape and thickness, use the felt wheels and cones, with pulverized pumice stone and water, and in a short time can have a polish almost equal to any of the metals.

In regard to the metal in the mouth, I find no fault; find only "the weight" by those who have worn the rubber. I have worn a piece seven months in my mouth, and can say this much, that the acids of the mouth have but little or no effect upon it. L. A. H.

